# The effect of anti-TNF drugs on the intestinal microbiota in patients with spondyloarthritis, rheumatoid arthritis, and inflammatory bowel diseases

**DOI:** 10.1515/rir-2024-0003

**Published:** 2024-03-31

**Authors:** Francesco Ciccia, Nikolas Konstantine Dussias, Saviana Gandolfo, Fernando Rizzello, Paolo Gionchetti

**Affiliations:** Rheumatology Unit, Department of Precision Medicine, Università degli Studi della Campania “L. Vanvitelli”, Naples, Italy; IBD Unit, IRCCS, Azienda Ospedaliero-Universitaria di Bologna, SSD Malattie Infiammatorie Croniche Intestinali, University of Bologna, Bologna Italy; Rheumatology Unit, Azienda Ospedaliera San Giovanni Bosco, Naples, Italy

**Keywords:** inflammatory bowel diseases, microbiota dysbiosis, rheumatoid arthritis, spondyloarthritis, tumor necrosis factor

## Abstract

Spondyloarthritis (SpA), rheumatoid arthritis (RA), and inflammatory bowel diseases (IBD) are chronic inflammatory autoimmune diseases that are associated with alterations in the composition of the intestinal microbiota (*i.e*., dysbiosis). For SpA and RA, a gut-joint-enthesis axis is hypothesized and recent data suggests that dysbiosis may contribute directly to initiating and perpetuating joint and spine inflammation. Biologic drugs targeting tumor necrosis factor (TNF) are effective in treating these diseases and have been shown to partially restore the disrupted microbiome. Hence, drugs that affect both the intestinal and joint components of these diseases, such as anti-TNF drugs, may act on the intestinal microbiome. However, despite the remarkable efficacy of anti-TNF-α treatments, non-responders are frequent, and predictors of patient outcomes have not been identified. In this narrative review, we summarize recent research on the downstream effects of anti-TNF drugs on the intestinal microbiota in SpA, RA, and IBD. We also discuss whether these changes could have a role as predictive biomarkers of anti-TNF response.

## Introduction

Spondyloarthritis (SpA), rheumatoid arthritis (RA), and inflammatory bowel diseases (IBD) are chronic inflammatory autoimmune diseases that cause considerable morbidity.

SpA is an umbrella term for a family of diseases that occur in the spine, peripheral joints, and entheses. The clinical manifestations of SpA are variable and are characterized by inflammation, pain, stiffness in the spine and pelvic joints, enthesitis, and the presence of human leukocyte antigen B27 (HLA-B27).^[1]^ SpA has a reported prevalence that ranges from 0.20% in South-East Asia to 1.61% in Northern Arctic communities.^[2]^

RA affects approximately 1% of the population worldwide, with a reported global prevalence of 460 cases per 100, 000 population between 1980 and 2019.^[3,4]^ The clinical manifestations of RA are heterogeneous and complex but are characterized by joint and synovial inflammation giving rise to the destruction of cartilage and bone elements of the joint, causing considerable pain and disability.^[3]^

IBD affects the gastrointestinal tract and exhibits a relapsing-remitting course. IBD is divided into two main types, ulcerative colitis (UC) and Crohn’s disease (CD), both causing irreversible damage to the structure and function of the gastrointestinal tract.^[5]^ IBD is prevalent worldwide. Although its incidence is stabilizing in the Western world, an increasing prevalence is reported due to its low mortality rate and young age of onset. Conversely, newly westernized countries in Asia, Africa, and South America report a rapidly increasing incidence of IBD, although its prevalence remains low in these societies.^[6]^

SpA is one of the more common extra-intestinal manifestations in the setting of IBD, and a shared pathogenesis underlying these diseases is emerging, including genetic predisposition, environmental factors, and pro-inflammatory cytokine pathways (*i.e*., tumor necrosis factor [TNF], interleukin [IL]-23, and IL-17).^[7,8,9,10]^ Conversely, the relationship between IBD and RA is less clear.^[11,12]^

TNF (also known as TNF-α) is a cytokine that regulates the inflammatory response and is involved in the pathogenesis of several inflammatory autoimmune diseases, including RA, SpA, and IBD.^[13,14]^ The development of biologics inhibiting TNF have been effective in the clinical treatment of these inflammatory autoimmune diseases.

Alterations in the composition of the intestinal microbiota (*i.e*., dysbiosis) have also been documented in patients with RA, SpA, and IBD, and it is of interest to understand whether biologic drugs used to treat these disorders also affect the intestinal microbiota.

In this narrative review, we summarize recent research on the downstream effects of anti-TNF drugs on the intestinal microbiota in SpA, RA, and IBD. We also discuss whether these changes could have a role as predictive biomarkers of anti-TNF response.

## Spondyloarthritis

The pathophysiology of SpA remains largely unknown. Although the association with genetic factors has been established for decades, recent data suggest an important role for the intestinal microbiome in driving innate and adaptive immune responses that may be relevant in initiating and perpetuating joint and spine inflammation.^[15]^ The high prevalence of subclinical intestinal inflammation in patients with SpA (approximately 60% of cases), together with accumulating data demonstrating the occurrence of gut dysbiosis in SpA patients, support this concept.^[16]^ In addition, assuming that SpA is a disease driven by a type 3 immune response, most of the IL-17 producing cells are represented by mucosal-derived cells such as the group 3 innate lymphoid cells (ILC3s), mucosal-associated invariant T (MAIT) cells, γδ T cells, and tissue-resident memory T (T_RM_) cells.^[15]^ This evidence, together with the demonstration of the intestinal origin of inflammatory cells found in peripheral blood, synovial fluid, and inflamed marrow of patients with SpA, support the hypothesis of the existence of a gut-joint-enthesis axis in SpA.^[16,17]^

In this hypothesis, the altered microbiome would interact with innate immune cells resident in the intestine, aberrantly activating them and promoting their extra-intestinal migration in the joint/spine of patients with SpA thereby promoting inflammation.^[18]^ This concept is also supported by evidence that, under germ-free conditions, SKG mice (an animal model of human autoimmune arthritis) and HLA-B27 TG rats (an animal model to assess the relative contribution of acquired and innate immune responses in SpA) do not develop joint inflammatory manifestations.^[19,20]^ Such evidence suggests that modifying the microbiome may represent a viable therapeutic strategy in patients with SpA and that, to some extent, the treatments we use may also act by affecting the composition of the gut microbiome. However, studies involving modification of the microbiome in patients with active SpA through the use of probiotics did not improve the signs or symptoms of the disease.^[21]^ More recently, studies to improve the intestinal microbiome and epithelial permeability using a gluten-free diet have been designed, and we await the results of this “intestinal-driven” approach on disease activity.^[22]^

For the past 20 years, however, biologic drugs effective in treating SpA have been used. It seems conceivable, therefore, that the use of drugs that are effective on both the intestinal and joint components of the disease, such as anti-TNF drugs, may modify the gut-joint-enthesis axis by acting on the intestinal microbiome. In this regard, Yin and colleagues^[23]^ demonstrated that anti-TNF therapy was correlated with the restoration of the disturbed microbiome in untreated patients with SpA to that of healthy controls. Moreover, an enrichment of bacterial peptides homologous to epitopes presented by HLA-B27 was observed in the stools of patients with SpA, suggesting either an inability of HLA-B27 to eliminate them or their involvement in driving HLA-B27-associated immune reactions. Anti-TNF therapy largely restored the disrupted microbiome in SpA-treated patients with a reduction in potentially arthritogenic bacterial peptides compared with untreated patients.^[23]^ Similar results have been reported in patients with enteropathic arthritis, with significant increases in the Lachnospiraceae family and Coprococcus genus identified in fecal samples after 6 months of anti-TNF therapy (both *P* < 0.05 *vs*. baseline).^[24]^ However, differences between anti-TNF responders and non-responders in terms of alpha (intrasample) and beta (inter-sample) diversity were not observed.

Despite the remarkable efficacy of anti-TNF-α treatments, non-responders are frequent, and predictors of patient outcomes have not been identified. In an exploratory study by Bazin and colleagues,^[25]^ the intestinal microbiota composition in SpA patients was not significantly modified after 3 months of anti-TNF-α treatment. However, a higher baseline percentage of Burkholderiales order was identified in responder patients, and the authors suggested that the presence of this taxonomic node could act as a biomarker to predict clinical response to anti-TNF-α treatment.

It is interesting to note how some studies, such as that of Manasson and colleagues,^[26]^ suggest a neutral effect of anti-TNF therapy on the intestinal microbiome compared with other treatments, including anti-IL-17. Indeed, significant changes in the abundance of specific taxa, specifically Clostridiales and Candida albicans, were reported after treatment with IL-17 inhibitors compared with TNF inhibitors. These alterations correlated with changes in bacterial community co-occurrence, metabolic pathways, IL-23/Th17-related cytokines, and various fatty acids. Moreover, ileal biopsies obtained from patients with SpA who developed Crohn’s disease while on anti-IL-17 therapy showed an expansion of type 2 immune cells producing IL-17/IL-25 and expansion of type-2 innate lymphoid cells (ILC2s), compared with pre-IL-17i treatment levels.^[26]^

## Rheumatoid Arthritis

The possibility that a gut-joint axis is also functional in patients with RA is a hypothesis that has gained popularity in recent years. Indeed, outcomes from pre-clinical and clinical studies suggest that intestinal dysbiosis occurs in patients with RA and is probably more relevant than the controversial dysbiosis of the oral cavity by the periodontal pathogen Porphyromonas gingivalis.^[27]^ In this regard, Maeda and colleagues^[28]^ demonstrated an intestinal microbiota dominated by Prevotella copri in a subpopulation of patients with early RA. Moreover, SKG mice harboring microbiota from RA patients had an increased number of intestinal Th17 cells and developed severe arthritis when treated with zymosan. The authors proposed that dysbiosis observed in patients with RA may contribute directly to the development of joint inflammation in the pathogenesis of RA.^[28]^

More recently, subclinical gut inflammation and altered intestinal permeability have been proven in RA patients, possibly depending on altered microbial composition, and restoration of normal intestinal permeability was associated with reduced arthritis severity in a collagen-induced arthritis model of RA.^[27]^

Based on these premises, it is conceivable that, even for RA, there is a concrete possibility that drugs routinely used for the treatment of joint diseases can also modify the composition of the microbiome and that this may represent an additive mechanism through which the clinical efficacy of the drug manifests. The study by Picchianti-Diamanti and colleagues ^[29]^ characterized the gut microbiota in RA patients who were either treatment-naïve or who had previously received treatment with methotrexate and/or etanercept. This study reported that the composition of the intestinal microbiota was altered in treatment-naïve RA patients compared with healthy controls. Intestinal dysbiosis in RA patients was also associated with several serological and clinical parameters. In particular, the phylum of Euryarchaeota was directly related to disease activity score and was identified as an independent risk factor. This study also showed, for the first time, that RA patients treated with the anti-TNF-α, etanercept, had partial restoration of a beneficial microbiota.^[29]^

## Inflammatory Bowel Disease: Crohn’s Disease and Ulcerative Colitis

In recent years, the medical treatment of patients with IBD has changed significantly. Novel therapeutic agents have become available, and our knowledge of how to optimize treatment strategies for IBD patients has improved markedly. Biologic therapies are now the mainstay of treatment in IBD, and antibodies targeting TNF-α have become essential in the armamentarium for the treatment of both UC and CD.

Current clinical guidelines recommend anti-TNF agents for patients who are refractory to other treatments.^[30,31,32]^ Currently, there are four anti-TNF agents available to treat IBD. These include infliximab ^[33,34]^ and adalimumab, ^[35,36]^ which are authorized for use in UC and CD in the European Union (EU) and the United States of America (USA), and golimumab, ^[37,38]^ which is authorized for use in UC in the EU and the USA. Certolizumab pegol^[39]^ has only been approved for treating CD in the USA and Switzerland, and will not be discussed here.

These anti-TNF drugs are effective at inducing symptom relief, disease remission, and mucosal healing in IBD patients.^[40,41]^ They have also been reported to reduce the need for surgery and hospitalizations among patients with moderate-to-severe IBD and contribute to better disease control with a reduction in (late) complications of the disease and improved quality of life.^[42]^

Unfortunately, a considerable proportion of patients fail to respond to anti-TNF induction therapy and are categorized as primary non-responders. In addition, up to 50% of patients who initially respond to anti-TNF therapy show a loss of response (LOR) over time (i. e., secondary LOR), which often leads to discontinuation of treatment.^[43]^

An important factor that contributes to secondary LOR is the development of anti-drug antibodies (ADA). This ‘immunogenicity’ can result in faster clearance of the drug subsequently leading to lower serum drug levels and LOR.^[44,45,46]^ The formation of ADA is also associated with infusion reactions.^[47]^ There may also be other reasons for the success or failure of anti-TNF therapy, such as differences in drug turnover, drug secretion, unknown genetic factors, and differences in disease progression.

In patients who experience LOR to a particular anti-TNF agent, dose escalation or intensification, either by increasing the dose or decreasing the dosing intervals, is commonly used as a rescue strategy to regain the therapeutic effect.^[48]^ Therefore, the need for individualized treatment regimens is becoming more important.

The phyla Bacteroidetes and Firmicutes dominate the gut microbiota of healthy humans.^[49]^ Conversely, CD and UC patients exhibit dysbiosis consisting of decreased proportions of Bacteroidetes and Firmicutes and increased proportions of microbial commensal communities, such as those in the phyla Actinobacteria and Proteobacteria, or elevation of specific pathogenic organisms, such as adherent-evasive Escherichia coli.^[50,51,52,53]^ As well as decreased bacterial diversity, patients with IBD also present with an increase in colonization of the mucosal surface compared with healthy subjects.^[50,51,52,53^]

One study showed that dysbiosis in the gut microbiome of IBD patients was characterized by changes in Firmicutes and Proteobacteria phyla.^[50]^ This study, which correlated the variation between the gut microbiome of IBD patients and a number of environmental factors, including treatment, also showed that both treatment and environmental factors were associated with independent changes in the gut microbiome. The authors concluded that although these perturbations in bacterial composition were modest, they were associated with major perturbations of gut microbiome function.

A separate study, which evaluated short-term changes in the intestinal microbiota of CD patients, identified a significant reduction in the prevalence of E. coli after 1 and 3 months of treatment with adalimumab compared with baseline levels and identified Firmicutes as the main phylogroup recovered after 3 months of treatment.^[54]^ The authors concluded that adalimumab induces short-term changes in the microbiota composition, which parallel the partial recovery of the gut bacterial ecology. They also suggested that quantitation of Faecalibacterium prausnitzii and E. coli, which are representative of dysbiosis, may be reliable indicators of the healing state of the intestinal mucosa.

The composition of the intestinal antimicrobial peptide expression and microbiota have also been suggested to influence treatment outcomes in patients with UC.^[55]^ Specifically, differential expression of antimicrobial peptides was identified in treatment responders and non-responders, with a higher abundance of F. prausnitzii and lower dysbiosis indexes identified at baseline in UC patients who responded to anti-TNF therapy (evaluated 12–14 weeks after baseline) compared with non-responders.

Moreover, a prospective, multicenter, observational study identified the F. prausnitzii/E. coli ratio as a reliable indicator of response to anti-TNF treatment.^[56]^ In this study, which assessed 27 CD patients initiating anti-TNF treatment, CD patients who responded to anti-TNF treatment showed partial restoration of the microbiome characteristic to that of healthy individuals (*i.e*., they presented a tendency towards eubiosis). F. prausnitzii and E. coli were identified as the main representatives of two genera strongly linked to intestinal eubiosis/dysbiosis in the context of inflammatory activity in CD. The F. prausnitzii/E. coli ratio was proposed as a noninvasive biomarker of therapeutic response to differentiate between anti-TNF responders and non-responders.^[56]^

Alatawi and colleagues ^[57]^ evaluated the possible influence of the gut microbiota in response to anti-TNF-α treatment in patients with IBD. Importantly, a difference in gut microbiome composition was observed in the fecal microbiota of responder and primary non-responder IBD patients. In IBD patients who demonstrated primary non-response to anti-TNF-α agents, dysbiosis was identified as a prominent feature of the microbiome. Aspects of dysbiosis were observed, which included decreased biodiversity, a lack of short-chain fatty acid (SCFA)-producing bacteria, and an increase in opportunistic pathogenic microbiota.^[57]^

Finally, a prospective, longitudinal cohort study that aimed to identify predictive biomarkers of anti-TNF response in CD reported a range of candidate biomarkers associated with primary non-response in CD.^[58]^ Specifically, metabolic profiles, with alterations in lipid metabolites, bile acids, and amino acid pathways, were predictive of primary non-response.

## Conclusion

Dysbiosis is reported in patients with inflammatory autoimmune diseases, including SpA, RA, and IBD. While these diseases are not correlated in terms of microbiota changes and there is no supporting evidence to suggest that the change of microbiota is related to disease severity or subtype, the role of the intestinal microbiome is of increasing interest. Importantly, anti-TNF therapy has been correlated with partial restoration of the disturbed microbiome in these patients. Not all patients respond to anti-TNF drugs, however, and identifying validated non-invasive biomarkers of therapeutic response is imperative. Some studies have proposed that microbial changes after biologic therapies may be able to differentiate between responders and non-responders, but this will require validation in large prospective cohorts. Overall, there is an urgent need to better understand the downstream effects of anti-TNF drugs on the intestinal microbiome in patients with SpA, RA, and IBD and whether these changes could be used as non-invasive biomarkers of treatment response.
